# 431. Title Implementing a Comprehensive Care Model as a Proposal for Long Covid Syndrome Care in a Latin American Country

**DOI:** 10.1093/ofid/ofad500.501

**Published:** 2023-11-27

**Authors:** Jaime Andres Pineda Capera, Carlos Alvarez, Nathali Carolina Gonzalez Alvarado, Juan Manuel Correa Hernandez, Paola Andrea Rengifo Bobadilla, Patrick Francois Tarquino Aparicio

**Affiliations:** Clinica Colsanitas, bogota, Distrito Capital de Bogota, Colombia; Departamento Enfermedades Infecciosas, Clínica Colsanitas, Universidad Nacional de Colombia, BOGOTA, Distrito Capital de Bogota, Colombia; Clinica Colsanitas, bogota, Distrito Capital de Bogota, Colombia; Clinica Colsanitas, bogota, Distrito Capital de Bogota, Colombia; Clinica Colsanitas, bogota, Distrito Capital de Bogota, Colombia; Clinica Colsanitas, bogota, Distrito Capital de Bogota, Colombia

## Abstract

**Background:**

Post-COVID-19 long syndrome or "LONG COVID" is defined by WHO criteria according to the Post-COVID Functional Score (PCFS) and Barthel scales and classified by the persistence of symptoms up to 12 weeks after initial infection. Through a survey conducted in a population of 33500 adults in Bogotá, Colombia, and the confirmatory of SARSCoV2 infection via (PCR or antigen test), we demonstrated 63% of people reported some persistent symptoms after three months of the acute event. With its high frequency, this new clinical condition makes a breach where comprehensive care centers for its management and rehabilitation were necessary. We designed an innovative, individualized, comprehensive transdisciplinary model to provide integral attention to patients diagnosed with Long COVID.

**Methods:**

Patients identified by the questionnaire as a Long COVID were scheduled for submission and care at the Center for Comprehensive Care Post Covid (CAIP). An integral evaluation included the assessment by several specialties, such as physiatry, physiotherapy, psychology, nutrition, nursing, social work, and internal medicine practitioners, according to an established attention model in our clinic (Fig 1).

**Figure 1.**
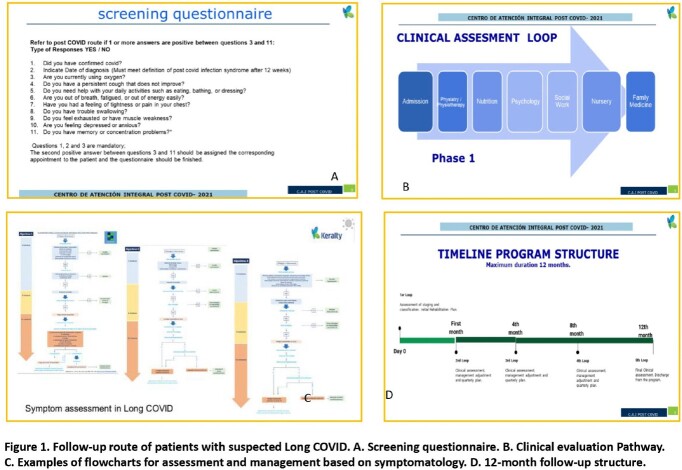
Follow up route of patients with suspected Long COVID. A. Screening questionnaire.

**Results:**

448 patients with long covid were screened between September 2022 and March 2023 in the CAIP. The average age of the population served was 52.6 years. The Barthel average score was 97.8. Patients classified as PCFS grade 1 were 282, 133 were grade 2, and 32 were grade 3. A total of 2230 physical and occupational therapy interventions were performed and 120 psychotherapy sessions with positive findings in functional and aerobic capacity between circuits (Fig 2). With the successful discharge of 50 patients, without remaining limitations to perform their basic activities, 48% were asymptomatic with PCFS: 0 and the remaining with PCFS 1, with a minimum symptomatic compromise.

**Figure:**
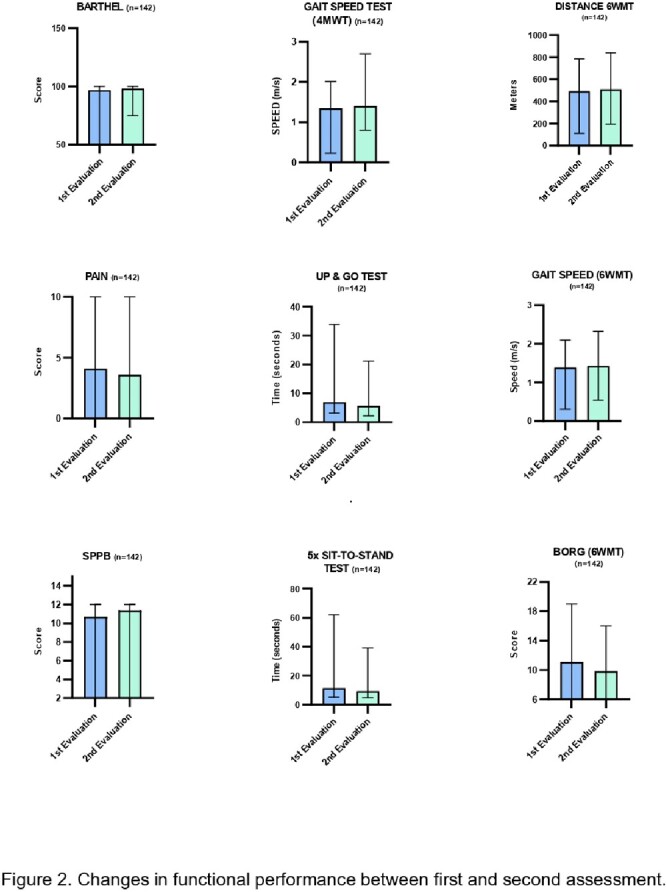

**Conclusion:**

There are governmental and clinical challenges to the comprehensive care of the population with disabilities related to Long COVID. CAIP, as a transdisciplinary health care and recovery model, which was born in response to long COVID patients, showed great effectiveness in return people great improvement in functionality to reincorporate into our society from the pandemic COVID19 in Colombia.

**Disclosures:**

**All Authors**: No reported disclosures

